# NCEH1 promotes breast cancer progression by regulating NRP1 and activating the TNF-α/NF-κB signalling pathway

**DOI:** 10.1080/19336918.2026.2616948

**Published:** 2026-01-27

**Authors:** Jie Sun, Yaqian Liu, Jieji Mo, Jialin Zhou, Xue Bai, Boshi Gu, Jun Li, Haidong Zhao

**Affiliations:** a Department of Breast Surgery, The Second Hospital of Dalian Medical University, Dalian, Liaoning, China; b Department of Thyroid, Breast and Head and Neck Surgery, Xinjiang Uygur Autonomous Region Hospital of Traditional Chinese Medicine, The Fourth Affiliated Hospital of Xinjiang Medical University, Urumqi Municipality, Xinjiang Province, China

**Keywords:** NCEH1, NRP1, TNF-α/NF-κB signalling pathway, breast cancer

## Abstract

**Purpose:**

Neutral cholesterol ester hydrolase 1 (NCEH1), a key enzyme in cellular lipid metabolism, is associated with cancer progression. Its molecular functions in breast cancer remain poorly understood.

**Methods:**

This study evaluated the expression of NCEH1 in breast cancer patients using multiple databases. Functionally, the effects of NCEH1 silencing or overexpression on breast cancer cell growth and motility were investigated. RNA-seq was employed to identify downstream target genes and signalling pathways.

**Results:**

The expression of NCEH1 in breast cancer tissues and cells was significantly higher than that in normal tissues and cells. Silencing NCEH1 suppressed breast cancer cell proliferation and migration. Mechanistically, NCEH1 regulated Neuropilin-1 (NRP1) expression, and both promoted malignant phenotypes in breast cancer by activating the TNF-α/NF-κB signalling pathway.

**Conclusion:**

Our findings demonstrate that NCEH1 accelerates breast cancer progression by modulating NRP1 and activating the TNF-α/NF-κB signalling pathway. Collectively, NCEH1 represents a potential novel biomarker and therapeutic target for breast cancer.

## Introduction

Breast cancer is a highly prevalent malignant tumour worldwide, characterized by high heterogeneity. Its incidence rate reaches 11.5%, ranking second only to lung cancer [1,2]. Although current treatments have expanded from traditional approaches such as local excision, chemotherapy, radiotherapy, and endocrine therapy to the field of targeted therapies, patients still face the challenge of tumour recurrence and metastasis [3]. Therefore, identifying suitable biomarkers for breast cancer is critical for elucidating its pathogenesis and improving survival rates.

Neutral cholesterol ester hydrolase 1 (NCEH1) is one of the key hydrolases in the lipid metabolic pathway in cells. NCEH1 is a serine hydrolase. The gene encoding NCEH1 is located on human chromosome 3. Structurally, its N-terminus is connected to the endoplasmic reticulum (ER) membrane, while the catalytic domain resides within the ER lumen. This structural characteristic allows it to play a catalytic role at specific locations within cells and participate in lipid metabolism processes [4].

According to a previous study, it has been found that NCEH1 can promote the hydrolysis of 2-acetylmonoalkyl glycerol ether to produce monoacylglyceride enzyme. Both are overexpressed in various cancer cells such as melanoma, pancreatic cancer, and ovarian cancer. The high expression is closely related to cancer cell proliferation, invasion, and other aggressive behaviours [5]. Recent studies have shown that NCEH1 is highly expressed in various malignant tumours. Single-cell RNA sequencing analyses demonstrate high NCEH1 expression in pancreatic cancer samples. This genetic marker serves as an independent prognostic factor and a biomarker for predicting chemoresistance and immune cell infiltration [6]. In addition, Tang et al. further identified NCEH1 as a prognostic predictor for gastric cancer patients and a marker of response to chemotherapy combined with immune checkpoint inhibitors through transcriptomic analysis of cholesterol metabolism genes [7]. Similarly, RNA sequencing in head and neck cancer epithelial-mesenchymal transition (EMT) revealed that an NCEH1-based gene risk score predicts overall survival (OS) [8].

Neuropilin-1 (NRP1) is a glycoprotein that plays critical roles in various processes, including angiogenesis, tumour malignant progression, and the regulation of immune system homeostasis [9,10]. Previous studies have demonstrated that NRP1 acts as a co-receptor binding multiple extracellular ligands. It not only promotes tumour angiogenesis by enhancing the vascular endothelial growth factor (VEGF) signalling pathway but also interacts with various ligands such as semaphorins and transforming growth factor β), thereby activating multiple downstream oncogenic signalling pathways and regulating tumour cell migration and invasion [11,12]. Multiple evidences indicate abnormal NRP1 expression and its carcinogenic effects in various malignancies [13–15]. For instance, overexpression of NRP1 in breast cancer accelerates tumour progression [16]. In non-small cell lung cancer cells, the expression level of NRP1 shows a synchronized increase with the expression of EMT-related proteins [17]. Furthermore, NRP1 influences the stability of regulatory T cells within the tumour microenvironment, participating in immune regulation [18].

It is well-known that TNF-α can accelerate malignant cancer progression by promoting cell proliferation, inhibiting apoptosis, and inducing angiogenesis [19]. NF-κB is a key regulatory molecule in the TNF-α signalling pathway [20,21]. Studies demonstrate that NF-κB mediates multiple processes such as angiogenesis, inflammatory responses, cell proliferation, metastasis, and tumour development in various eukaryotic cells [22]. In glioblastoma cells, inhibition of NF-κB activity significantly downregulates the expression of the pro-angiogenic factor VEGF [23]. The NF-κB signalling pathway plays a significant role in the development and metastasis of breast cancer [24]. Frequent overactivation of this pathway creates a favourable microenvironment for tumour growth [25]. It regulates the expression of various molecules associated with cell invasion, promotes EMT, and accelerates the metastatic process [26].

Given its overexpression in multiple cancers and strong association with tumour progression, NCEH1 may represent a potential therapeutic target. We hypothesize that NCEH1 may play a critical role in breast cancer. This study aims to elucidate its biological functions and mechanisms in breast cancer. Our findings demonstrate that the expression level of NCEH1 in breast cancer is significantly increased. NCEH1 can promote the proliferation and migration of breast cancer cells and participate in the regulation of EMT. We further demonstrated that NCEH1 can affect NRP1 expression and promote breast cancer progression through the TNF-α/NF-κB signalling pathway.

## Methods

### Bioinformatics analysis

The expression of NCEH1 in tumour and normal tissues was analysed using the GEPIA2 platform (http://gepia2.cancer-pku.cn/) [27] based on the Genotype-Tissue Expression (GTEx) and the Cancer Genome Atlas (TCGA) databases. The criteria are as follows: log2 Fold change = 1 and *p* value ≤0.01. The Kaplan–Meier Plotter database (https://kmplot.com/analysis/) [28] was employed to evaluate the correlation between NCEH1 expression and relapse-free survival (RFS) or distant metastasis-free survival (DMFS) in breast cancer patients. The UALCAN database (http://ualcan.path.uab.edu) [29] assessed the association of NCEH1 with axillary lymph node metastasis. TIMER 2.0 (https://cistrome.shinyapps.io/timer/) [30] was used to analyse the correlation between NCEH1 and NRP1 expression, visualized as scatter plots with a significance threshold of *p* valve set to less than 0.05.

### Cell lines

Breast cancer cell lines (MDA-MB-231, MDA-MB-453, HCC1806, HCC1954, MCF-7, SK-BR3), human mammary epithelial cells (MCF-10A), and HEK293T cells were obtained from the American Type Culture Collection (ATCC). MCF-10A cells were cultured in mammary epithelial cell-specific medium (LONZA, CC-3151). MDA-MB-231, MCF-7, SK-BR3, and HEK293T cells were cultured in DMEM (GIBCO, C11995500BT), HCC1806 and HCC1954 cells were cultured in RPMI 1640 (GIBCO, C11875500BT), and MDA-MB-453 cell was cultured in L-15 (Solarbio, LA9510), all supplemented with 10% fetal bovine serum (FBS) (VivaCell, C04001-500), 100 μg/mL streptomycin, and 100 U/mL penicillin (NCM Biotech, C100c5). All cells were cultured at 37°C in a humidified incubator with 5% CO2.

### siRNA transfection

MDA-MB-231 and MCF-7 cells at 50% confluence in 6-well plates were transfected with specific small interfering RNA (siRNAs) targeting NCEH1 (si-NCEH1-1, 5’-GGCUAGUUCCAAAGGUUUATT-3′, 5′-UAAACCUUUGGAACUAGCCTT-3′. si-NCEH1-2, 5′-CCGGACUAGGAAUAGUUACAUTT-3′, 5′-AUGUAACUAUUCCUAGUCCGGTT-3’.) or nonspecific siRNA (Sangon Biotech (Shanghai) Co., Ltd.) using Lipofectamine 2000 (Invitrogen, 11,668,019). Each siRNA and transfection reagent were diluted in serum-free medium (GIBCO, 31,985,070), mixed and incubated for 20 min before adding to cells. Forty-eight hours after transfection, knockdown efficiency was validated via qPCR and Western blotting.

### Cell viability and colony formation assays

Cell viability and colony formation assays used specific siRNA or overexpressing vectors to transfect breast cancer cells. For viability assays, transfected MDA-MB-231 and MCF-7 cells were seeded in 96-well plates. At indicated time points, 10 μL of Cell Counting Kit-8 (CCK8) (NCM Biotech, C6005) reagent was added per well, incubated for 2–4 h, and the optical density (OD) at 450 nm was measured using a Bio-Rad microplate reader. For colony formation assays, transfected cells were harvested and re-seeded into 6-well plates and cultured for 7–14 days until cell colonies appeared. Cell colonies were fixed in methanol for 10 min, stained with 0.1% crystal violet (Solarbio, C8470) at room temperature, protected from light for 15 min, photographed and counted.

### Scratch wound healing and Transwell migration assays

Scratch wound healing and Transwell migration assays used specific siRNA or overexpressing vectors to infect breast cancer cells. For scratch assays, transfected MDA-MB-231 and MCF-7 cells were scratched by using a sterile 200 μL pipette tip in 6-well plates until cell fusion reached 80–90%. Cells were washed and cultured in serum-free medium. The scratched areas were photographed using a microscope at 0 and 24 h, respectively. For the Transwell migration assay, the transfected cells were resuspended in 100 μL of serum-free medium and added to the upper chamber, while the lower chamber contained 600 μL of complete medium with 10% FBS in a 24-well plate. After 24 h, cells were fixed in methanol for 10 min and stained with 0.1% crystal violet for 15 min, protected from light. Wipe it clean and use a microscope (Leica) to image.

### Clinical samples

Samples of breast tumours and adjacent tissues were collected from patients diagnosed with breast cancer at the Second Affiliated Hospital of Dalian Medical University. Tissues were collected and stored in a refrigerator at −80°C.

All operations in this study were carried out in accordance with the Declaration of Helsinki. All participating patients have signed informed consent forms. The study protocol was approved by the Institutional Ethics Committee (Project Registration Number: KY2025-114–01).

### Western blotting

Cells were lysed in Radio Immunoprecipitation Assay (RIPA) (Beyotime, P0013B) buffer with protease and phosphatase inhibitors. After thorough lysis and centrifugation, the protein concentration of the supernatant was measured using the bicinchoninic acid (BCA) protein detection kit (TIANGEN, PA115-02). Proteins were separated by sodium dodecyl sulfate-polyacrylamide gel electrophoresis (SDS-PAGE), transferred to nitrocellulose (NC) membranes (Milipore, HATF08130), and blocked with 5% skim milk 1 h. Then the primary antibody was incubated overnight at 4°C. The main antibodies used in this study include NCEH1 (1:1000, Solarbio), NRP1 (1:1000, Proteintech)、E-cadherin (1:1000, Cell Signaling Technology), N-cadherin (1:1000, Cell Signaling Technology), Vimentin (1:1000, Cell Signaling Technology), β-catenin (1:1000, Cell Signaling Technology), Slug (1:1000, Cell Signaling Technology), Snail (1:1000, Cell Signaling Technology), TNF-α (1:1000, Wanleibio), NF-κB (1:1000, Wanleibio), p-NF-κB (1:1000, Wanleibio), IκBα (1:1000, Wanleibio), p-IκBα (1:1000, Wanleibio), GAPDH (1:5000, Wanleibio). After washing, the NC membrane was incubated with the corresponding secondary antibody at room temperature. Western blotting was detected using enhanced chemiluminescence.

### RNA extraction and qPCR

Total RNA was extracted with TRIzol reagent (Takara, RNAiso Plus T9108) according to the manufacturer’s instructions and reverse-transcribed using MonScript™ RTIII All-in One Mix (Monad, MR05101M). qPCR was performed with Hieff UNICON SYBR Green Master Mix (Yeasen, 11184ES08). The primers used in this experiment included NCEH1 (forward 5′-CCATTGAATACAGGCTAGTTCC-3′, reverse 5′-CAAATTCTGCCTGGATCAACC-3′), NRP1 (forward 5′-CCCAACAGCCTTGAATGCAC-3′, reverse 5′-ATTTCTAGCCGGTCGTAGCG-3′), and GAPDH (forward 5′-CTGGGCTACACTGAGCACC-3′, reverse 5′-AAGTGGTCGTTGAGGGCAATG-3′).

### Lentiviral transfection

The target plasmids NCEH1 shRNA, NCEH1 overexpression (OE), NRP1 OE and packaging plasmids ps.PAX2 and pMD2.G were mixed with Polyethylenimine (PEI) (Yeasen, 40816ES01) and allowed to stand at room temperature for 20 min. The mixture was added to a HEK293T petri dish to prepare virus fluid. The obtained virus solution was mixed with the culture medium, and 8 μg/mL polybrene (Yeasen, 40804ES76) was added to infect MB-MDA-231 and MCF-7 cells. After screening with puromycin (MedChemExpress, HY-B1743), stably transfected cells were obtained.

### Immunohistochemistry (IHC)

Tumour tissue was fixed overnight in 10% formalin for IHC. Embed in wax and cut into 4 µm slices. Tissue sections were deparaffinized, rehydrated, and stained with antibodies against NCEH1 (1:100, Solarbio), NRP1 (1:100, Proteintech), or Ki67 (1:100, Abcam) using standard protocols (ZSGB-BIO, PV-9000).

### Immunofluorescence (IF)

MDA-MB-231 and MCF-7 cells were seeded on cell slides. Fixed with 4% paraformaldehyde. Cells were permeabilized with 0.5% TritonX-100 (Solarbio, IT9100). After that, NCEH1 antibody (1:50, Solarbio) was used overnight at 4°C, and after the incubation with fluorescent secondary antibody (1:500, Proteintech), the coverslip was stained with DAPI (Solarbio, S2110) to counterstain the cell nucleus. The location of NCEH1 protein in MDA-MB-231 and MCF-7 cells was observed using a microscope.

### Cell cycle, apoptosis, and senescence assays

All experiments were performed 48 h after cell transfection. For cell cycle experiment, transfected cells were fixed overnight at 4°C using 70% ethanol, stained with propidium iodide (KGI Biosciences, KGA9101-100), and detected by flow cytometry. For the apoptosis experiment, cell precipitates were collected after digestion with EDTA free of trypsin, resuspended and stained using Annexin V-FITC/PI staining (KGI Biosciences, KGA1101-100). The apoptosis results were detected by flow cytometry. For the senescence assays, the cell aging kit (Beyotime, C0602) was used to stain the cells according to the instructions, and then the staining was observed using a microscope.

### Hematoxylin-eosin staining (H&E staining)

Paraffin sections of mouse tissues were first baked at 60°C for 2 h. After dewaxing and rehydration of the paraffin sections, the cell nuclei were stained with hematoxylin staining solution, followed by staining of the cytoplasm with eosin staining solution (Solarbio, G1120). After the sections were dried, they were mounted and preserved with neutral balsam.

### Animal studies

The animal experimental protocol was conducted in accordance with the animal license agreement approved by the Research Committee of Dalian Medical University (Registration Number: AEE24134). Healthy 4-week-old female BALB/c nude mice were selected and purchased from SPF (Beijing) Biotechnology Co., Ltd. Mice were numbered and randomly assigned to groups using a randomization tool. For the in vivo proliferation assay, female BALB/c nude mice (*n* = 20) were randomly divided into four groups (sh-NC, NRP1-OE, sh-NCEH1, sh-NCEH1 + NRP1-OE, with five mice in each group). MDA-MB-231 cells were injected subcutaneously into the axilla of mice at a dose of 5 × 10^6^ cells. Five days after injection, the tumour size was recorded every 5 days using a vernier caliper, and the tumour volume was calculated as V=(length × width^2^)/2. When tumours in the control group reached approximately 1 cm, the animals were euthanized, the tumour was excised and photographed. For the in vivo metastasis assay, female BALB/c nude mice (*n* = 20) were randomly divided into four groups (sh-NC, NRP1-OE, sh-NCEH1, sh-NCEH1 + NRP1-OE, with five mice in each group). After tail vein injection of MDA-MB-231 cells at a dose of 5 × 10^6^ cells into the mice, the animals were euthanized 3 months post-injection. The lung tissues were harvested for paraffin-embedded for subsequent analysis.

### Statistical analysis

Data were analysed and charted using GraphPad Prism 9.0. Experimental data are expressed as mean ± standard deviation (SD). Two group comparisons were analysed by Student’s *t*-test, and multiple group comparisons were analysed by analysis of variance (ANOVA). *p* < .05 was considered statistically significant.

## Results

### Differential expression of NCEH1 in tumours and its clinical prognostic relevance

**Figure 1.**
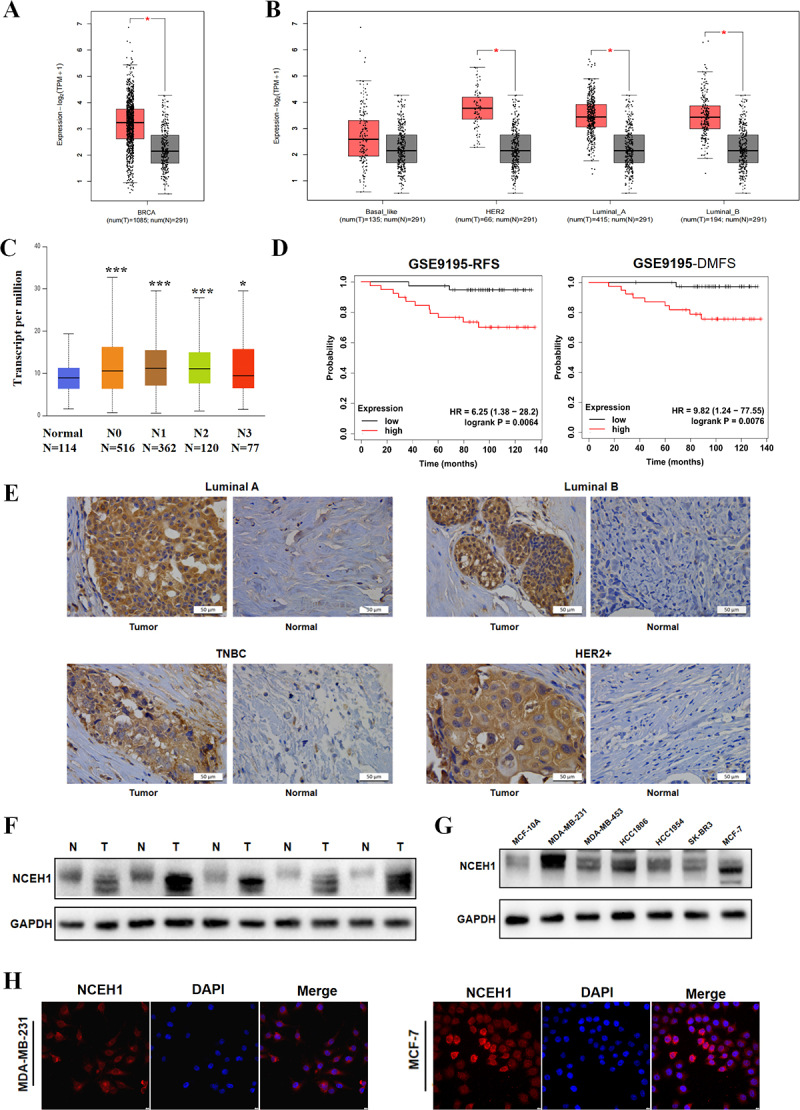
NCEH1 is highly expressed in breast cancer tissues and cell lines.

Analysis of public databases revealed significantly elevated NCEH1 expression in breast cancer tissues compared to normal tissues (Figure 1(A)). In different types of breast cancer, the expression level of NCEH1 in tumour tissues was higher than that in normal tissues (Figure 1(B)). UALCAN database analysis demonstrated a significant association between high NCEH1 expression and lymph node metastasis (Figure 1(C)). In addition, survival analysis from the Kaplan–Meier Plotter database showed that NCEH1 expression levels were significantly correlated with patient prognosis, and breast cancer patients with high NCEH1 expression had shorter RFS and DMFS (Figure 1(D)). Bioinformatics analysis showed that the high expression of NCEH1 is linked to malignant progression and poor prognosis in breast cancer.

(A) NCEH1 expression in breast cancer and adjacent normal tissues (BRCA, Breast Cancer). (B) NCEH1 expression in different molecular subtypes of breast cancer. (C) UALCAN database analysis of NCEH1 expression correlation with lymph node metastasis. (D) Kaplan–Meier survival analysis of NCEH1 expression levels and prognosis in breast cancer patients. (RFS: relapse-free survival; DMFS: distant metastasis-free survival). (E) Immunohistochemistry (IHC) showed NCEH1 expression in different molecular subtypes of breast cancer (scale bar = 50 μm). (F) Western blotting of NCEH1 protein levels in tumour and adjacent normal tissues (N: Normal; T: Tumour). (G) Western blotting of NCEH1 expression in normal mammary epithelial cells (MCF-10A) and breast cancer cell lines. (H) Immunofluorescence localization of NCEH1 in MDA-MB-231 and MCF-7 cells (scale bar = 10 μm).

Alt Text. Multi-dimensional analyses demonstrating that NCEH1 is highly expressed in breast cancer tissues and cell lines, including expression profiling across subtypes, correlation with clinical outcomes, and subcellular localization.

(A) A box plot shows that the mRNA expression level of NCEH1 is significantly higher in breast cancer tissues compared to adjacent normal tissues. (B) A box plot compares the mRNA expression of NCEH1 across different molecular subtypes (e.g., Luminal, HER2+, Basal-like) of breast cancer. (C) The box plot analysed by the UALCAN database shows a significant association between high expression of NCEH1 and lymph node metastasis in breast cancer patients. (D) Two Kaplan–Meier survival curves indicate that patients with high NCEH1 expression have significantly shorter relapse-free survival and distant metastasis-free survival than those with low expression. (E) Four immunohistochemical micrographs show prominent NCEH1 expression in Luminal A, Luminal B, triple-negative breast cancer (TNBC), and HER2+ subtypes, with predominant cytoplasmic localization (scale bar = 50 μm). (F) A Western blot image comparing NCEH1 protein levels in paired tumour and adjacent normal tissues from the same patient, the expression of NCEH1 increased significantly compared to the adjacent tissues. (G) A Western blot image shows low NCEH1 protein expression in normal mammary epithelial cells (MCF-10A) and elevated expression in multiple breast cancer cell lines (MDA-MB-231, MDA-MB-453, HCC1806, HCC1954, SK-BR3, MCF-7). (H) Two sets of confocal microscopy images: immunofluorescence staining of MDA-MB-231 and MCF-7 cells. Red fluorescence (NCEH1) is primarily localized in the cytoplasm, and blue indicates the nuclei (scale bar = 10 μm).

In order to verify the clinical applicability of bioinformatics analysis results, we detected and localized the expression of NCEH1 in clinical breast cancer tissue samples. IHC showed prominent NCEH1 expression in Luminal A, Luminal B, triple-negative breast cancer (TNBC), and HER2+ subtypes, with predominant cytoplasmic localization (Figure 1(E)). Western blotting results showed that in breast cancer tissues, the expression of NCEH1 increased significantly compared to the adjacent tissues (Figure 1(F)). Additionally, NCEH1 was overexpressed in breast cancer cell lines (MDA-MB-231, MDA-MB-453, HCC1806, HCC1954, SK-BR3, MCF-7) relative to normal mammary epithelial cells (MCF-10A) (Figure 1(G)). We selected triple negative breast cancer cells MDA-MB-231 and Luminal breast cancer cells MCF-7 with relatively high expression levels as the next experimental cells. To further understand the distribution of NCEH1, we conducted immunofluorescence experiments in MDA-MD-231 and MCF-7 cells, and we found that NCEH1 was mainly localized in the cytoplasm of tumour cells (Figure 1(H)). Collectively, these results suggest that NCEH1 is highly expressed in breast cancer and may be involved in the occurrence and development of breast cancer.

### NCEH1 promotes breast cancer cell growth and migration

**Figure 2.**
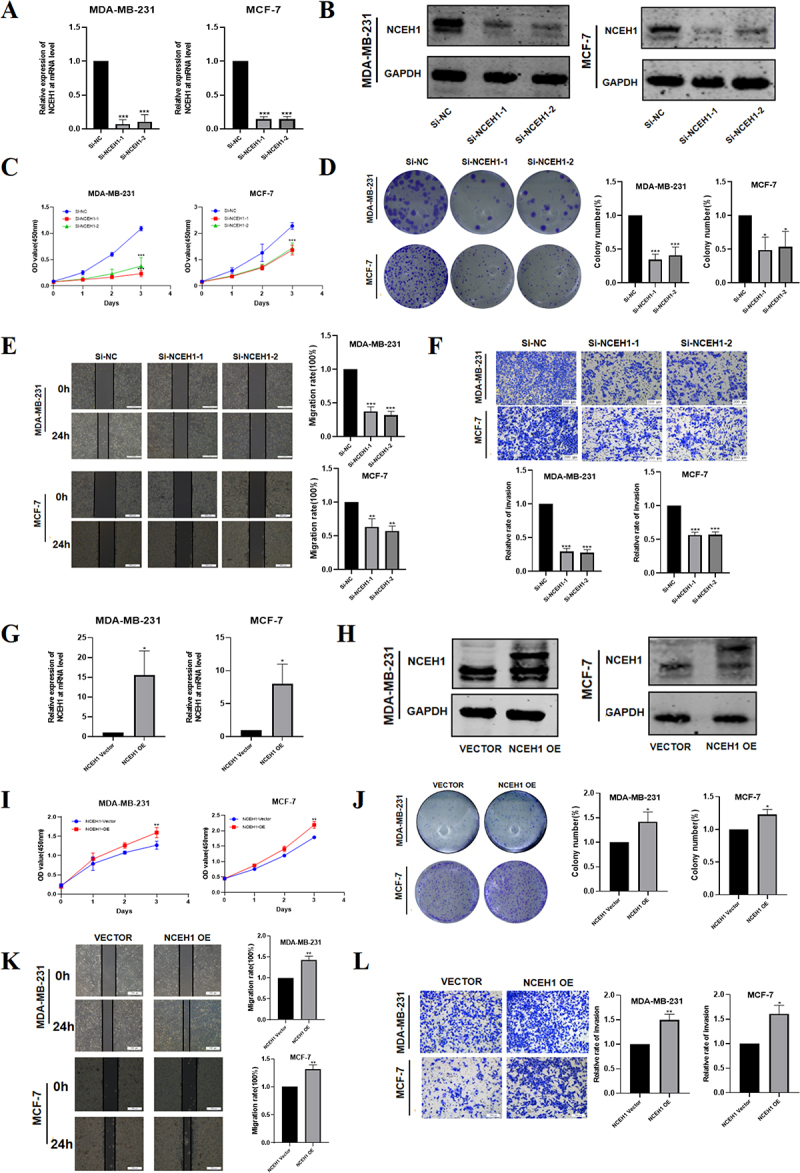
NCEH1 promotes breast cancer cell proliferation and migration.

To investigate the functional role of NCEH1, we silenced its expression using NCEH1-specific siRNAs. qPCR (Figure 2(A)) and Western blotting (Figure 2(B)) demonstrated successful transfection of NCEH1 siRNA and significant knockdown of NCEH1 expression. NCEH1 silencing significantly inhibited the proliferation ability (Figure 2(C)) and colony formation ability (Figure 2(D)) in MDA-MB-231 and MCF-7 cells. It shows that it has a promoting effect on the growth of breast cancer.

(A) qPCR analysis of NCEH1 mRNA levels in MDA-MB-231 and MCF-7 cells transfected with NCEH1-specific siRNAs (si-NCEH1-1, si-NCEH1-2). (B) Western blotting of NCEH1 protein levels in MDA-MB-231 and MCF-7 cells transfected with NCEH1-specific siRNAs (si-NCEH1-1, si-NCEH1-2). (C) CCK-8 assay measured the relative viability of MDA-MB-231 and MCF-7 cells NCEH1 knockdown. (D) Colony formation assay examined the effect of NCEH1 knockdown on the cloning ability of MDA-MB-231 and MCF-7 cells. (E) Scratch wound healing assay measured the changes in migration ability of MDA-MB-231 and MCF-7 cells after NCEH1 knockdown (scale bar = 500 μm). (F) Transwell migration assay analysed the effect of NCEH1 knockdown on the migration ability of MDA-MB-231 and MCF-7 cells (scale bar = 200 μm). (G) qPCR analysis of NCEH1 mRNA levels in MDA-MB-231 and MCF-7 cells treated with NCEH1 that overexpresses lentivirus. (H) Western blotting analysis of NCEH1 protein levels in MDA-MB-231 and MCF-7 cells treated with NCEH1 that overexpresses lentivirus. (I) After NCEH1 overexpression, cell viability in MDA-MB-231 and MCF-7 cells was measured by CCK-8 assay. (J) Colony formation assay examined the effect of NCEH1 overexpression on the cloning ability of MDA-MB-231 and MCF-7 cells. (K) Scratch wound healing assay was used to determine the effect of NCEH1 overexpression on the migration ability of MDA-MB-231 and MCF-7 cells (scale bar = 500 μm). (L) Transwell migration assay to analyse the effect of NCEH1 overexpression on the migration ability of MDA-MB-231 and MCF-7 cells (scale bar = 200 μm). Data represented mean ± SD of three independent experiments. The level of significance was indicated by ***p  < 0.001, **p  < 0.01, *p  < 0.05.

In order to explore the migration ability of NCEH1 in breast cancer, we conducted scratch wound healing assays. After silencing NCEH1, MDA-MB-231 and MCF-7 cells inhibited scratch healing for a certain period of time and slowed down the speed (Figure 2(E)). Transwell migration assay showed that silencing NCEH1 significantly reduced the migration ability of MDA-MB-231 and MCF-7 cells (Figure 2(F)). The above results suggest that NCEH1 can accelerate the progression of breast cancer and promote tumour migration.

In order to demonstrate the carcinogenic effect of NCEH1 in breast cancer cell lines, we transfected lentivirus fluids overexpressing NCEH1 into MDA-MB-231 and MCF-7 cells. NCEH1 overexpression efficiency was demonstrated by qPCR (Figure 2(G)) and Western blotting (Figure 2(H)). Our results demonstrated that overexpression of NCEH1 significantly increased the viability (Figure 2(I)) and colony forming ability (Figure 2(J)) of MDA-MB-231 and MCF-7 cells and promoted the migration ability of cells (Figures 2K and 2L). The above results further support that NCEH1 promotes the proliferation and migration capabilities of breast cancer cells and accelerates the progression of breast cancer.

### NCEH1 modulates cell cycle, apoptosis, and senescence

**Figure 3.**
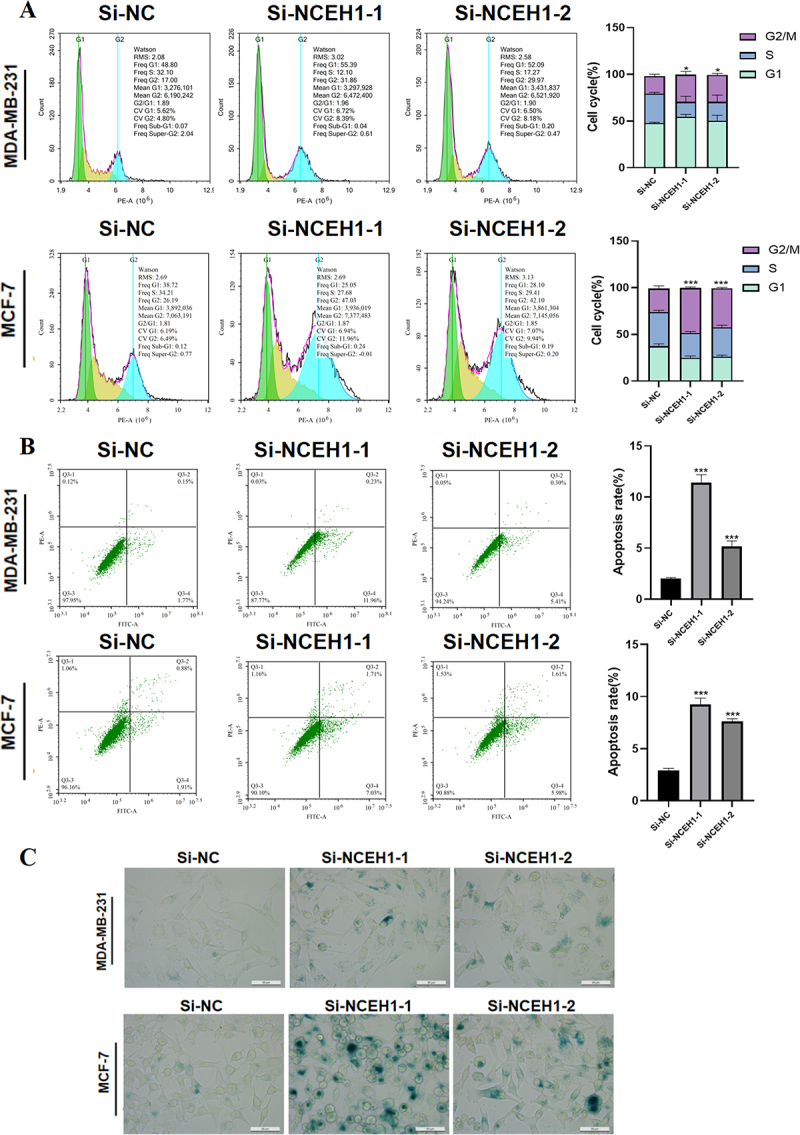
NCEH1 regulates cell cycle, apoptosis, and senescence.

Based on the mentioned effect of NCEH1 on the proliferation of breast cancer cells, we conducted cell cycle assays and found that NCEH1 silencing induced G2/M phase arrest in MDA-MB-231 and MCF-7 cells (Figure 3(A)). When cells enter the G2/M arrest state, it will inhibit cell mitosis, delay cell proliferation, and is not conducive to DNA damage repair. Apoptosis assays demonstrated that NCEH1 silencing increased the number of early and late apoptosis of tumour cells, promoting apoptosis of breast cancer cells (Figure 3(B)). Furthermore, senescence-associated β-galactosidase activity was elevated in silenced cells (Figure 3(C)), indicating enhanced cellular senescence.

(A) Cell cycle assay was used to measure the changes in the ratio of MDA-MB-231 and MCF-7 cells after NCEH1 knockdown. (B) Flow apoptosis assay was used to measure the effect of NCEH1 knockdown on apoptosis in MDA-MB-231 and MCF-7 cells. (C) Senescence assay was used to measure the effect of NCEH1 knockdown on the aging of MDA-MB-231 and MCF-7 cells. Data represented mean ± SD of three independent experiments. The level of significance was indicated by ***p  < 0.001, **p  < 0.01, *p  < 0.05.

### NCEH1 regulates epithelial-mesenchymal transition (EMT)

**Figure 4.**
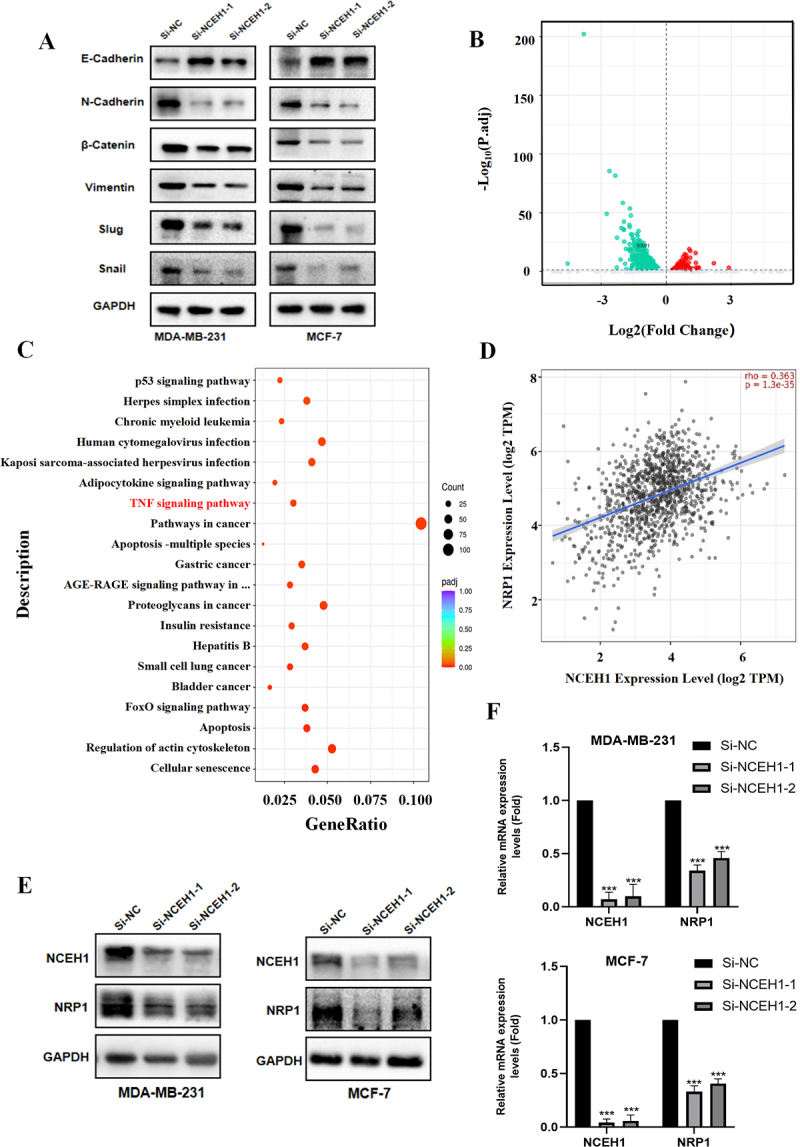
NCEH1 affects EMT and downstream target identification in breast cancer.

EMT refers to the process in which epithelial cells undergo a series of changes and eventually transform into cells with mesenchymal characteristics [31]. Abnormal activation of EMT changes cell characteristics, enhances migration and invasion capabilities, anti-apoptotic properties, and promotes extracellular matrix degradation [32]. Based on the results of phenotyping experiments of NCEH1 in breast cancer cells, high expression of NCEH1 can promote the proliferation and migration of cells, and knockdown of NCEH1 can promote the apoptosis of breast cancer cells. We speculate that NCEH1 may promote tumour progression through EMT. We found that after NCEH1 knockdown, the expression of E-cadherin increased in MDA-MB-231 and MCF-7 cells, while the expression levels of N-cadherin, β-catenin, and Vimentin all decreased, and the mesenchymal characteristics of the cells were weakened. In addition, the expression levels of Slug and Snail proteins, key transcription factors promoting EMT, were also significantly reduced (Figure 4(A)). These results demonstrated that NCEH1 knockdown can inhibit EMT and help inhibit cell metastasis and invasion capabilities. This implies that NCEH1 drives EMT to enhance metastatic potential.

(A) Western blotting measured the expression changes of related protein levels in EMT after NCEH1 knockdown in MDA-MB-231 and MCF-7. (B) Volcanic map of up-regulated and down-regulated genes in NCEH1 siRNA transfected MDA-MB-231 cells and control cells (SiNC). (C) KEGG pathway enrichment analysis of the down-regulated gene set after NCEH1 gene knockdown in MDA-MB-231 cells. (D) Correlation between NCEH1 and NRP1 expression in breast cancer. (E) Western Blotting measured the protein expression level of NRP1 after NCEH1 knockdown in MDA-MB-231 and MCF-7 cells. (F) qPCR was used to detect the mRNA expression level of NRP1 after NCEH1 knockdown in MDA-MB-231 and MCF-7 cells. Data represented mean ± SD of three independent experiments. The level of significance was indicated by ***p  < 0.001, **p  < 0.01, *p  < 0.05.

### NCEH1 promotes tumour growth via NRP1 regulation

**Figure 5.**
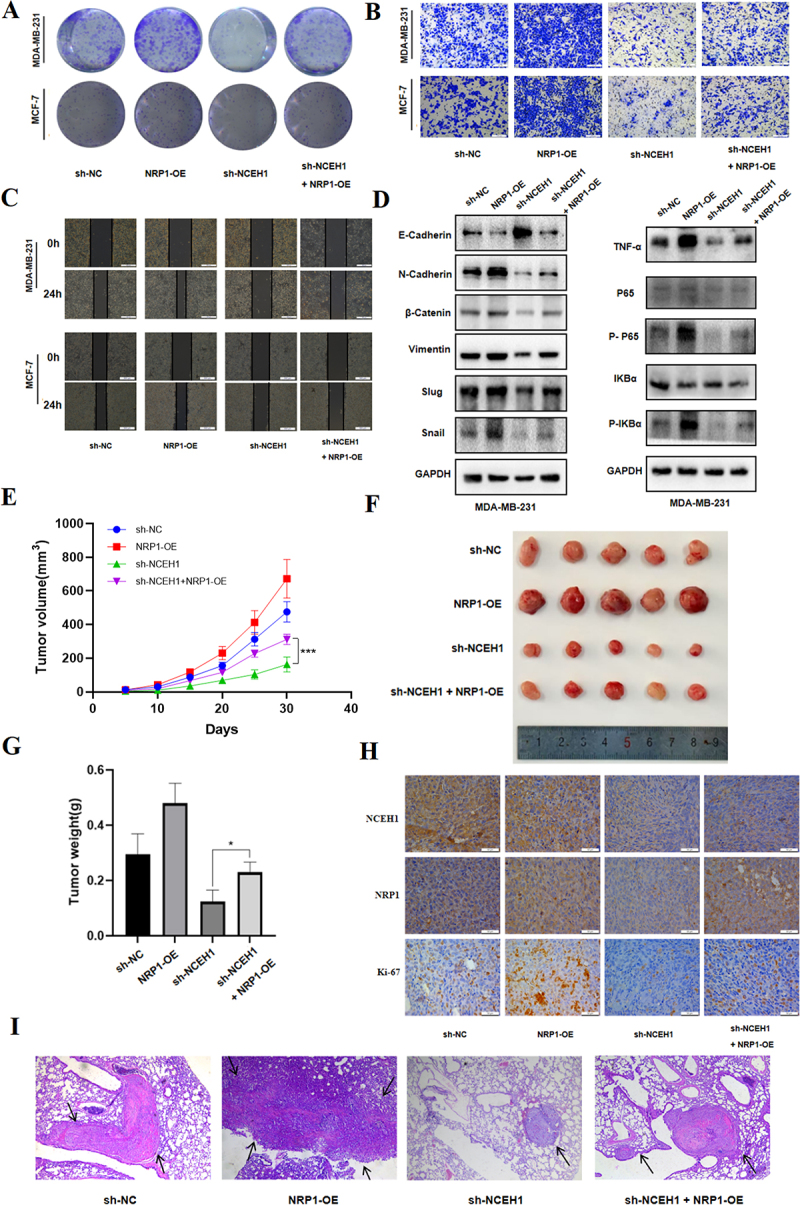
NCEH1 regulates the progression of breast cancer cells by targeting NRP1.

To further analyse the potential molecular mechanism by NCEH1 promotes breast cancer progression, we analysed the transcriptome changes after silencing NCEH1 expression in MDA-MB-231 (Figure 4(B)). In addition, KEGG enrichment analysis of differentially expressed genes showed that NCEH1 silencing affects a range of signalling pathways in breast cancer cells (Figure 4(C)). Because in the early stage, we verified that NCEH1 can promote the occurrence and development of breast cancer through EMT. Through a large number of literature review, neuropilin 1 (NRP1) was screened among differential genes. NRP1 is a unique transmembrane glycoprotein that plays a role in promoting tumour progression, accelerating angiogenesis, and regulating immune responses. NRP1 has been shown to be an effector molecule that mediates EMT and accelerates tumour progression through EMT in a variety of tumours [33–35]. Notably, NCEH1 expression positively correlated with NRP1 levels in breast cancer (Figure 4(D)). Therefore, we speculate that NCEH1 may promote the malignant progression of breast cancer by affecting the expression of NRP1. It was determined by qPCR and Western blotting analysis that silencing of NCEH1 reduced NRP1 mRNA and protein levels (Figures 4E and 4F). We simultaneously silenced NCEH1 and overexpressed NRP1 in breast cancer cells to analyze their impact on the malignant phenotype of the cells. Our results showed that NCEH1 silencing significantly inhibited cell proliferation and migration compared to control groups, while these effects were partially reversed after NRP1 overexpression (Figures 5A–5C).

(A) Colony formation assay in MDA-MB-231 and MCF-7 cells co-transfected with NCEH1 shRNA and NRP1 OE. (B) Transwell migration assay showed the effect of co- transfected with NCEH1 shRNA and NRP1 OE on the migration ability of MDA-MB-231 and MCF-7 cells. (C) Scratch wound healing assay confirmed the effect of co-transfected with NCEH1 shRNA and NRP1 OE on the migration ability of MDA-MB-231 and MCF-7 cells. (D) Western blotting of TNF-α/NF-κB pathway and EMT-related proteins in cells with NCEH1 knockdown and NRP1 OE. (E-G) MDA-MB-231 cells that stably knocked out NCEH1 and overexpressed NRP1 were injected subcutaneously into mice. The tumour volume within 30 days after injection of the tumour was calculated (E), the tumour image at the end of the experiment was displayed (F), the weight of the tumour after removal from the mice was weighed (G). (H) Representative photos showing IHC staining of NCEH1, NRP1, and Ki-67 in tumour tissue (scale bar = 50 μm). (I) Representative H&E staining of lung metastatic nodules after tail vein injection of tumour cells in mice (scale bar = 500 μm). Data represented mean ± SD of three independent experiments. The level of significance was indicated by ***p  < 0.001, **p  < 0.01, *p  < 0.05.

Abnormal activation of TNF signalling pathway is closely related to tumour growth, invasion, metastasis and angiogenesis [36]. TNF can promote the proliferation and survival of tumour cells by activating transcription factors such as NF-κB, and inhibit apoptosis of tumour cells [22]. In addition, studies have shown that NRP1, as a co-receptor for TNF-α, can promote the expression of NF-κB under stimulation from TNF-α [37]. RenD, XuJ and Kim et al. have reported that in different malignant tumours, the NF-κB pathway can regulate EMT and promote cancer progression [38–40]. Mechanistically, we found that NCEH1 knockdown suppressed TNF-α/NF-κB signalling, while NRP1 overexpression restored pathway activation (Figure 5(D)). In addition, inhibition of NCEH1 and overexpression of NRP1 reversed the inhibitory effect of knockdown of NCEH1 on EMT (Figure 5(D)). Similarly, in vivo experiments with breast cancer cells with stable knockdown NCEH1 and NRP1 overexpression showed that NRP1 overexpression partially alleviated the tumour growth inhibition caused by NCEH1 knockdown (Figures 5E–5G). IHC staining of the formed xenografts further demonstrated that overexpression of NRP1 salvaged the tumour growth inhibition caused by NCEH1 (Figure 5(H)). In vivo experiments on lung metastasis of breast cancer cells demonstrated that breast cancer cells with stable NCEH1 knockdown and NRP1 overexpression also showed that NRP1 overexpression partially alleviated the NCEH1-mediated inhibitory effect on tumour metastasis caused by NCEH1 knockdown (Figure 5(I)). These results support the hypothesis that NCEH1 promotes breast cancer growth and metastasis through NRP1 expression.

## Discussion

Breast cancer is the most common female malignant tumour worldwide, seriously endangering women’s physical and mental health [41]. While advancements in early detection and treatment have reduced mortality rates, the heterogeneity and complexity of breast cancer often lead to poor prognosis and diminished quality of life, presenting ongoing challenges for patients [42,43]. Overall, the epidemiological profile of breast cancer reflects at least in part our limitations in understanding the molecular signalling pathways of tumourigenesis and the functions of many genes in cancer [44,45]. Therefore, elucidating the signalling mechanisms underlying breast cancer initiation and progression may reveal novel therapeutic strategies and targets.

NCEH1 is one of the important hydrolases in the lipid metabolic pathway in cells. The gene encoding NCEH1 is located on the q arm of chromosome 3, a chromosomal region that is highly amplified in various malignant tumours [46]. Notably, the reliability of NCEH1 as a therapeutic target is supported by its consistent carcinogenic effects observed in multiple malignant tumours. Growing evidence indicates that NCEH1 is abnormally overexpressed in various cancer type and is closely associated with poor prognosis. Jessani N et al. first found that NCEH1 is positively correlated with the proliferation ability and invasiveness of malignant tumour cells [5]. In addition, literature has shown that the expression of NCEH1 is significantly increased in various cancers such as pancreatic cancer, gastric cancer and prostate cancer and is closely related to the degree of local invasion and lymph node metastasis of the tumour [47–49]. NCEH1 is highly expressed in pancreatic cancer samples, participating in cancer progression and chemoresistance to gemcitabine, suggesting its potential as a target for efficacy evaluation and resistance reversal in pancreatic cancer [6]. In cholangiocarcinoma, serum and tissue cholesterol levels are significantly elevated, promoting tumour development. Aurora kinase B (AURKB) can increase intratumoral cholesterol levels by regulating NCEH1, modulating lipid metabolism to drive tumour growth [50]. Furthermore, analysis of public datasets for breast cancer and major depressive disorder (MDD) showed significant upregulation of NCEH1, correlating with poor prognosis in breast cancer patients and the progression of MDD [51]. These studies collectively demonstrate that NCEH1 maintains a oncogenic function across multiple tumour types, providing strong evidence for its reliability as a therapeutic target in breast cancer treatment.

We have identified NCEH1 as an important biomarker and therapeutic target in breast cancer and have described a new mechanism for NCEH1 to promote breast cancer progression by affecting NRP1 expression and mediating the TNF-α/NF-κB signalling pathway. This may be the first report on the carcinogenic ability and possible molecular mechanisms of NCEH1 in the development of breast cancer.

Through bioinformatics analysis, we determined the expression profile of NCEH1 in breast cancer and found that high expression of NCEH1 is associated with poor prognosis. Clinical validation confirmed these findings, with immunohistochemistry and Western blotting demonstrating upregulated NCEH1 protein levels in tumours. In order to further explore the potential mechanism of NCEH1 in breast cancer, we conducted experiments and found that knockdown of NCEH1 significantly inhibited the proliferation and migration of breast cancer cells, indicating that NCEH1 plays a key role in regulating the malignant phenotype of breast cancer.

In terms of molecular mechanisms, we found that NCEH1 knockdown induced G2/M phase arrest and apoptosis while promoting cellular senescence. Cell cycle disorder is one of the hallmarks of cancer. Since the G2/M phase is the late stage of DNA synthesis and the G2/M checkpoint is the last checkpoint of cell mitosis, most tumour cells have defects in the G1/S checkpoint [52]. The cell cycle is arrested in the G2/M phase, which is not conducive to the repair of DNA damage, thereby passing the damaged DNA to daughter cells, which in turn causes apoptosis [53]. Cell aging is characterized by decreased cell proliferation ability and decline in physiological functions. After knocking down NCEH1, the proliferation rate of cancer cells slows down and may enter a stable non-dividing state, thereby inhibiting tumour growth and metastasis.

EMT refers to the biological process of transition from an epithelial to a mesenchymal phenotype [54]. Abnormal activation of EMT by tumour cells leads to increased invasiveness and mobility, which is considered a key event in tumour progression [55]. Our research shows that NCEH1 can mediate EMT, thereby participating in tumour adhesion and invasion.

Considering the important role of NCEH1 in EMT and the differentially expressed genes and signal transduction pathways based on RNA-Seq results. We explored the effect of NCEH1 on NRP1 regulation and its relationship with the TNF signalling pathway. NRP1 is widely expressed in tumour blood vessels and plays a role in promoting tumour progression, accelerating angiogenesis, and regulating immune responses through interaction with multiple regulatory factors [56]. NRP1 has been shown to be an effector molecule mediating EMT [57]. Studies have shown that NRP1 is up-regulated in cervical cancer, is related to disease progression and affects EMT [33]. NRP1 promotes the migration of liver cancer cells through EMT [34] and is also involved in TGF-β1-induced EMT in non-small cell lung cancer [35]. Abnormal activation of TNF signalling pathway is closely related to tumour growth, invasion, metastasis and angiogenesis. TNF mainly includes TNF-α and TNF-β, of which TNF-α is deeply researched and closely related to many diseases. TNF-α binds to TNF receptors (TNFR) on the cell surface, which in turn activates NF-κB [58]. Thereby promoting the proliferation and survival of tumour cells and inhibiting tumour cell apoptosis. It can also accelerate angiogenesis and promote the migration of tumour cells. TNF-α can promote EMT-related tumour invasion [59]. In different malignant tumours, the NF-κB pathway can regulate EMT and promote cancer progression [38–40,60]. NRP1 is a key factor in the TNF-α pathway and promotes the expression of NF-κB [37]. Combined with our findings that NCEH1 positively regulates NRP1 at both the mRNA and protein levels in breast cancer cells, and NRP1 overexpression significantly reverses the inhibitory effect caused by NCEH1 silencing. We fully believe that NCEH1 activates NRP1 to regulate the TNF-α/NF-κB signalling pathway and promotes breast cancer progression.

As a key downstream target of NCEH1, NRP1 plays an important role in promoting the progress of breast cancer. Based on our existing experimental evidence and relevant published studies, we have conducted an in-depth analysis of the potential mechanism by which NCEH1 regulates NRP1 transcription. As a neutral cholesterol ester hydrolase, NCEH1 primarily hydrolyses intracellular neutral cholesterol esters to release free cholesterol (FC). Cholesterol is a key component of the cell membrane, directly influences membrane fluidity and the structure and function of lipid rafts [61]. The signal transduction of many receptor tyrosine kinases, such as Vascular Endothelial Growth Factor Receptor 2 (VEGFR2), Epidermal Growth Factor Receptor (EGFR), relies on lipid rafts [62]. NRP1, as a co-receptor for VEGFR2, enhances complex stability through interaction [63]. Altered NCEH1 activity might affect the composition and function of lipid rafts, indirectly influencing the stability or signalling of NRP1, thereby providing feedback regulation on its transcriptional control.

Research has found that intracellular cholesterol can directly bind to Hypoxia Inducible Factor-1α (HIF-1α), enhancing its stability. Elevated intracellular cholesterol levels inhibit HIF-1α ubiquitination and degradation, thereby increasing its activity and transcriptional function [64]. Furthermore, changes in NCEH1 can lead to metabolic reprogramming, such as endoplasmic reticulum stress and reactive oxygen species level alterations, indirectly affecting HIF-1α regulation. HIF-1α has been confirmed to directly bind to the NRP1 promoter region located between positions −2009 and −2017. Studies have shown that HIF-1α upregulates NRP1 expression, promoting vasculogenic mimicry formation in lung adenocarcinoma and contributing to malignant progression [65]. Additionally, research in prostate cancer cells indicates that HIF1α binds to a specific region of the NRP1 promoter, regulating its transcriptional activation. NRP1 also interacts with EGFR, further activating the AKT signalling pathway, thereby promoting malignant progression in prostate cancer [18]. Therefore, we propose that NCEH1 may indirectly affect NRP1 transcription by regulating intracellular lipid homeostasis and signalling pathways.

Our current research suggests that NCEH1 could serve as a potential biomarker and therapeutic target for breast cancer. Of course, our research also has some limitations. In order to deeply explore the cancer-promoting role of NCEH1 in breast cancer, more research is needed in larger, multi-center breast cancer patients in the future. We expect that NCEH1 will become a new clinical prognostic and treatment indicator. More importantly, the regulatory relationship between NCEH1 and NRP1 still needs further experiments to verify. Given the complexity of tumour transcription, we need to further study whether NCEH1 cooperates with other factors or opposes the regulation of other breast cancer-related genes. Furthermore, this study has only validated the anti-tumour effects of NCEH1 intervention at cellular and animal levels, without initiating research on NCEH1-targeted drug development or combination therapies. Future work could focus on screening NCEH1-targeted inhibitors and exploring combination treatment strategies, thereby providing more comprehensive evidence to support the clinical translation of this therapeutic target.

In summary, this study demonstrates that NCEH1 can affect the transcription of NRP1 and thus upregulate its expression level. Both of them promote breast cancer proliferation and migration and other malignant phenotypes by activating the TNF-α/NF-κB signalling pathway and promote the progress of breast cancer.

## Conclusion

In conclusion, this study proves that NCEH1 is a key gene in breast cancer and it can affect NRP1 expression and promote breast cancer progression through activation of the TNF-α/NF-κB signalling pathway. This discovery marks the possibility that NCEH1 may become a new biomarker for breast cancer and a key target for therapeutic intervention.

## Data Availability

The datasets generated during or analysed during the current study are available from the corresponding author on reasonable request.

## References

[1] Ganesan K, Xu C, Wu S, et al. Ononin inhibits tumor bone metastasis and osteoclastogenesis by targeting mitogen-activated protein kinase pathway in breast cancer. Research. 2024;7:0553. doi: 10.34133/research.0553 39687715 PMC11648741

[2] Arnold M, Morgan E, Rumgay H, et al. Current and future burden of breast cancer: global statistics for 2020 and 2040. Breast. 2022;66:15–19. doi: 10.1016/j.breast.2022.08.010 36084384 PMC9465273

[3] Loibl S, Poortmans P, Morrow M, et al. Epidemiology and risk factors. Breast Cancer Lancet. 2021;397(10286):1750–1769. doi: 10.1016/S0140-6736(20)32381-3 33812473

[4] Igarashi M, Osuga J-I, Isshiki M, et al. Targeting of neutral cholesterol ester hydrolase to the endoplasmic reticulum via its N-terminal sequence[s]. J Lipid Res. 2010;51(2):274–285. doi: 10.1194/jlr.M900201-JLR200 19592704 PMC2803229

[5] Jessani N, Liu Y, Humphrey M, et al. Enzyme activity profiles of the secreted and membrane proteome that depict cancer cell invasiveness. Proc Natl Acad Sci. 2002;99(16):10335–10340. doi: 10.1073/pnas.162187599 12149457 PMC124915

[6] Chen J, Liu Z, Wu Z, et al. Identification of a chemoresistance-related prognostic gene signature by comprehensive analysis and experimental validation in pancreatic cancer. Front Oncol. 2023;13:1132424. doi: 10.3389/fonc.2023.1132424 37251940 PMC10213255

[7] Tang W, Li G, Lin Q, et al. Multiplex immunohistochemistry defines two cholesterol metabolism patterns predicting immunotherapeutic outcomes in gastric cancer. J Transl Med. 2023;21(1):887. doi: 10.1186/s12967-023-04758-4 38062450 PMC10702056

[8] Schinke H, Shi E, Lin Z, et al. A transcriptomic map of EGFR-induced epithelial-to-mesenchymal transition identifies prognostic and therapeutic targets for head and neck cancer. Mol Cancer. 2022;21(1):178. doi: 10.1186/s12943-022-01646-1 36076232 PMC9454230

[9] Vivekanandhan S, Mukhopadhyay D. Genetic status of KRAS influences transforming growth factor-beta (TGF-β) signaling: an insight into Neuropilin-1 (NRP1) mediated tumorigenesis. Semin Cancer Biol. 2019;54:72–79. doi: 10.1016/j.semcancer.2018.01.014 29409705 PMC6072630

[10] Pellet-Many C, Frankel P, Jia H, et al. Neuropilins: structure, function and role in disease. Biochem J. 2008;411(2):211–226. doi: 10.1042/BJ20071639 18363553

[11] Campos-Mora M, Morales RA, Gajardo T, et al. Neuropilin-1 in transplantation tolerance. Front Immunol. 2013;4:2013. doi: 10.3389/fimmu.2013.00405 PMC3839227 24324469

[12] Zachary IC. How neuropilin-1 regulates receptor tyrosine kinase signalling: the knowns and known unknowns. Biochem Soc Trans. 2011;39(6):1583–1591. doi: 10.1042/BST20110697 22103491

[13] Casazza A, Laoui D, Wenes M, et al. Impeding macrophage entry into hypoxic tumor areas by sema3a/nrp1 signaling blockade inhibits angiogenesis and restores antitumor immunity. Cancer Cell. 2013;24(6):695–709. doi: 10.1016/j.ccr.2013.11.007 24332039

[14] Jin Q, Ren Q, Chang X, et al. Neuropilin-1 predicts poor prognosis and promotes tumor metastasis through epithelial-mesenchymal transition in gastric cancer. J Cancer. 2021;12(12):3648–3659. doi: 10.7150/jca.52851 33995640 PMC8120182

[15] Snuderl M, Batista A, Kirkpatrick Nathaniel D, et al. Targeting placental growth factor/neuropilin 1 pathway inhibits growth and spread of medulloblastoma. Cell. 2013;152(5):1065–1076. doi: 10.1016/j.cell.2013.01.036 23452854 PMC3587980

[16] Tang YH, Rockstroh A, Sokolowski KA, et al. Neuropilin-1 is over-expressed in claudin-low breast cancer and promotes tumor progression through acquisition of stem cell characteristics and ras/mapk pathway activation. Breast Cancer Res. 2022;24(1):8. doi: 10.1186/s13058-022-01501-7 35078508 PMC8787892

[17] Wang M, Yi J, Gao H, et al. Radiation-induced YAP/TEAD4 binding confers non-small cell lung cancer radioresistance via promoting NRP1 transcription. Cell Death Dis. 2024;15(8):619. doi: 10.1038/s41419-024-07017-6 39187525 PMC11347582

[18] Zhang P, Chen L, Zhou F, et al. Nrp1 promotes prostate cancer progression via modulating EGFR-dependent AKT pathway activation. Cell Death Dis. 2023;14(2):159. doi: 10.1038/s41419-023-05696-1 36841806 PMC9958327

[19] Wang Y, Xu J, Zhang X, et al. Tnf-α-induced LRG1 promotes angiogenesis and mesenchymal stem cell migration in the subchondral bone during osteoarthritis. Cell Death Dis. 2017;8(3):e2715–e2715. doi: 10.1038/cddis.2017.129 28358372 PMC5386532

[20] Kim M, Jung K, Kim I-S, et al. Tnf-α induces human neural progenitor cell survival after oxygen–glucose deprivation by activating the NF-κB pathway. Exp Mol Med. 2018;50(4):1–14. doi: 10.1038/s12276-018-0033-1 PMC5938012 29622770

[21] Remels AHV, Gosker HR, Verhees KJP, et al. Tnf-α-induced NF-κB activation stimulates skeletal muscle glycolytic metabolism through activation of HIF-1α. Endocrinology. 2015;156(5):1770–1781. doi: 10.1210/en.2014-1591 25710281

[22] Bakshi HA, Quinn GA, Nasef MM, et al. Crocin inhibits angiogenesis and metastasis in colon cancer via TNF-α/NF-kB/VEGF pathways. Cells. 2022;11(9):1502. doi: 10.3390/cells11091502 35563808 PMC9104358

[23] Xie T-X, Xia Z, Zhang N, et al. Constitutive NF-κB activity regulates the expression of VEGF and IL-8 and tumor angiogenesis of human glioblastoma. Oncol Rep. 2010;23(3):725–732. doi: 10.3892/or_00000690 20127012

[24] Zhang J, Huang Z, Song C, et al. Prmt1-mediated parp1 methylation drives lung metastasis and chemoresistance via p65 activation in triple-negative breast cancer. Research. 2025;8:0854. doi: 10.34133/research.0854 40927753 PMC12415337

[25] Guo Q, Jin Y, Chen X, et al. Nf-κb in biology and targeted therapy: new insights and translational implications. Signal Transduct Target Ther. 2024;9(1):53. doi: 10.1038/s41392-024-01757-9 38433280 PMC10910037

[26] Mirzaei S, Saghari S, Bassiri F, et al. Nf-κb as a regulator of cancer metastasis and therapy response: a focus on epithelial–mesenchymal transition. J Cell Physiol. 2022;237(7):2770–2795. doi: 10.1002/jcp.30759 35561232

[27] Tang Z, Kang B, Li C, et al. Gepia2: an enhanced web server for large-scale expression profiling and interactive analysis. Nucleic Acids Res. 2019;47(W1):W556–W560. doi: 10.1093/nar/gkz430 31114875 PMC6602440

[28] Nagy Á, Lánczky A, Menyhárt O, et al. Validation of miRNA prognostic power in hepatocellular carcinoma using expression data of independent datasets. Sci Rep. 2018;8(1):9227. doi: 10.1038/s41598-018-27521-y 29907753 PMC6003936

[29] Chandrashekar DS, Bashel B, Balasubramanya SAH, et al. Ualcan: a portal for facilitating tumor subgroup gene expression and survival analyses. Neoplasia. 2017;19(8):649–658. doi: 10.1016/j.neo.2017.05.002 28732212 PMC5516091

[30] Li T, Fu J, Zeng Z, et al. Timer2.0 for analysis of tumor-infiltrating immune cells. Nucleic Acids Res. 2020;48(W1):W509–W514. doi: 10.1093/nar/gkaa407 32442275 PMC7319575

[31] Chen T, Zhou L, Li H, et al. Fatty acid synthase affects expression of ErbB receptors in epithelial to mesenchymal transition of breast cancer cells and invasive ductal carcinoma. Oncol Lett. 2017;14(5):5934–5946. doi: 10.3892/ol.2017.6954 29113229 PMC5661422

[32] Hu X, Jiang C, Hu N, et al. Adamts1 induces epithelial-mesenchymal transition pathway in non-small cell lung cancer by regulating TGF-β. Aging (Albany NY). 2023;15(6):2097–2114. doi: 10.18632/aging.204594 36947712 PMC10085599

[33] Gui Z, Ye Y, Li Y, et al. Construction of a novel cancer-associated fibroblast-related signature to predict clinical outcome and immune response in cervical cancer. Transl Oncol. 2024;46:102001. doi: 10.1016/j.tranon.2024.102001 38850798 PMC11214323

[34] Li X, Zhou Y, Hu J, et al. Loss of neuropilin1 inhibits liver cancer stem cells population and blocks metastasis in hepatocellular carcinoma via epithelial-mesenchymal transition. Neoplasma. 2021;68(2):325–333. doi: 10.4149/neo_2020_200914N982 33350850

[35] Ding Z, Du W, Lei Z, et al. Neuropilin 1 modulates tgf‑β1‑induced epithelial‑mesenchymal transition in non‑small cell lung cancer. Int J Oncol. 2020;56(2):531–543. doi: 10.3892/ijo.2019.4938 31894269 PMC6959462

[36] Li C, Wang F, Cui L, et al. Association between abnormal lipid metabolism and tumor. Front Endocrinol. 2023;14:2023. doi: 10.3389/fendo.2023.1134154 PMC10248433 37305043

[37] Li Y, Wang Z, Xu H, et al. Targeting the transmembrane cytokine co-receptor neuropilin-1 in distal tubules improves renal injury and fibrosis. Nat Commun. 2024;15(1):5731. doi: 10.1038/s41467-024-50121-6 38977708 PMC11231174

[38] Ren D, Yang Q, Dai Y, et al. Oncogenic mir-210-3p promotes prostate cancer cell emt and bone metastasis via NF-κB signaling pathway. Mol Cancer. 2017;16(1):117. doi: 10.1186/s12943-017-0688-6 28693582 PMC5504657

[39] Xu J, Zhang Z, Qian M, et al. Cullin-7 (CUL7) is overexpressed in glioma cells and promotes tumorigenesis via NF-κB activation. J Experim Clin Cancer Res. 2020;39(1):59. doi: 10.1186/s13046-020-01553-7 PMC7132976 32252802

[40] Kim NY, Jung YY, Yang MH, et al. Isoimperatorin down-regulates epithelial mesenchymal transition through modulating NF-κB signaling and CXCR4 expression in colorectal and hepatocellular carcinoma cells. Cell Signal. 2022;99:110433. doi: 10.1016/j.cellsig.2022.110433 35934221

[41] Barzaman K, Karami J, Zarei Z, et al. Breast cancer: biology, biomarkers, and treatments. Int Immunopharmacol. 2020;84:106535. doi: 10.1016/j.intimp.2020.106535 32361569

[42] Luis G, Godfroid A, Nishiumi S, et al. Tumor resistance to ferroptosis driven by stearoyl-CoA desaturase-1 (SCD1) in cancer cells and fatty acid binding protein-4 (FABP4) in tumor microenvironment promote tumor recurrence. Redox Biol. 2021;43:102006. doi: 10.1016/j.redox.2021.102006 34030117 PMC8163990

[43] Falchook G, Infante J, Arkenau H-T, et al. First-in-human study of the safety, pharmacokinetics, and pharmacodynamics of first-in-class fatty acid synthase inhibitor TVB-2640 alone and with a taxane in advanced tumors. eClinicalmedicine. 2021;34. doi: 10.1016/j.eclinm.2021.100797 PMC8040281 33870151

[44] Golemis EA, Scheet P, Beck TN, et al. Molecular mechanisms of the preventable causes of cancer in the United States. Genes Dev. 2018;32(13–14):868–902. doi: 10.1101/gad.314849.118 29945886 PMC6075032

[45] Hahn WC, Bader JS, Braun TP, et al. An expanded universe of cancer targets. Cell. 2021;184(5):1142–1155. doi: 10.1016/j.cell.2021.02.020 33667368 PMC8066437

[46] Song T, Yao M, Yang Y, et al. Integrative identification by Hi-C revealed distinct advanced structural variations in lung adenocarcinoma tissue. Phenomics. 2023;3(4):390–407. doi: 10.1007/s43657-023-00103-3 37589026 PMC10425312

[47] Lu Y, Zhang L, Chen X, et al. Nceh1 may be a prognostic biomarker for pancreatic cancer. Int J Clin Exp Pathol. 2020;13(11):2746–2752. 33284889 PMC7716126

[48] Xiao Y, Xie J, Liu L, et al. Nad(p)-dependent steroid dehydrogenase-like protein and neutral cholesterol ester hydrolase 1 serve as novel markers for early detection of gastric cancer identified using quantitative proteomics. J Clin Lab Anal. 2021;35(2):e23652. doi: 10.1002/jcla.23652 33219617 PMC7891516

[49] Raftopulos NL, Washaya TC, Niederprüm A, et al. Prostate cancer cell proliferation is influenced by LDL-cholesterol availability and cholesteryl ester turnover. Cancer Metabol. 2022;10(1):1. doi: 10.1186/s40170-021-00278-1 PMC8760736 35033184

[50] Liu F, Chen W, Zhang Z, et al. Targeting aurora kinase B regulates cholesterol metabolism and enhances chemoimmunotherapy in cholangiocarcinoma. Gut. 2025:gutjnl-2025–335291. doi: 10.1136/gutjnl-2025-335291 PMC12911660 40744724

[51] Xie H, Ding C, Li Q, et al. Identification of shared gene signatures in major depressive disorder and triple-negative breast cancer. BMC Psychiatry. 2024;24(1):369. doi: 10.1186/s12888-024-05795-z 38755543 PMC11100035

[52] Gemble S, Wardenaar R, Keuper K, et al. Genetic instability from a single S phase after whole-genome duplication. Nature. 2022;604(7904):146–151. doi: 10.1038/s41586-022-04578-4 35355016 PMC8986533

[53] Otifi HM, Alshyarba M, Fayi MA, et al. Computational docking and in vitro analysis identifies novel arylidene analogue fpmxy-14 against renal cancer cells by attenuating AKT. Oncol Res. 2021;29(3):217–227. doi: 10.32604/or.2022.03570 37304673 PMC10208040

[54] Georgakopoulos-Soares I, Chartoumpekis DV, Kyriazopoulou V, et al. Emt factors and metabolic pathways in cancer. Front Oncol. 2020;10. doi: 10.3389/fonc.2020.00499 PMC7154126 32318352

[55] Yang J, Antin P, Berx G, et al. Guidelines and definitions for research on epithelial–mesenchymal transition. Nat Rev Mol Cell Biol. 2020;21(6):341–352. doi: 10.1038/s41580-020-0237-9 32300252 PMC7250738

[56] Liu Y, Lu J. Mechanism and clinical application of thymosin in the treatment of lung cancer. Front Immunol. 2023;14. doi: 10.3389/fimmu.2023.1237978 PMC10493777 37701432

[57] Lengra Nd J, Pastushenko I, Vanuytven S, et al. Pharmacological targeting of netrin-1 inhibits emt in cancer. Nature. 2023;620(7973):402–408. doi: 10.1038/s41586-023-06372-2 37532929 PMC7615210

[58] Wang Y, Liu J, Huang B, et al. Mathematical modeling and application of IL-1β/TNF signaling pathway in regulating chondrocyte apoptosis. Front Cell Dev Biol. 2023;Volume 11. doi: 10.3389/fcell.2023.1288431 PMC10652750 38020878

[59] Li C-W, Xia W, Huo L, et al. Epithelial–mesenchymal transition induced by TNF-α requires NF-κB–mediated transcriptional upregulation of TWIST1. Cancer Res. 2012;72(5):1290–1300. doi: 10.1158/0008-5472.CAN-11-3123 22253230 PMC3350107

[60] Yang H-L, Thiyagarajan V, Shen P-C, et al. Anti-EMT properties of CoQ0 attributed to PI3K/Akt/NFκB/MMP-9 signaling pathway through ROS-mediated apoptosis. J Experim Clin Cancer Res. 2019;38(1):186. doi: 10.1186/s13046-019-1196-x PMC6505074 31068208

[61] Wu J, Ji P, Zhang A, et al. Impact of cholesterol homeostasis within cochlear cells on auditory development and hearing loss. Front Cell Neurosci. 2024;17. doi: 10.3389/fncel.2023.1308028 PMC10794501 38239289

[62] Pruis IJ, van Dongen GAMS, Veldhuijzen van Zanten SEM. The added value of diagnostic and theranostic PET imaging for the treatment of CNS tumors. Int J Mol Sci. 2020;21(3):1029. doi: 10.3390/ijms21031029 32033160 PMC7037158

[63] Lee C, Kim M-J, Kumar A, et al. Vascular endothelial growth factor signaling in health and disease: from molecular mechanisms to therapeutic perspectives. Signal Transduct Target Ther. 2025;10(1):170. doi: 10.1038/s41392-025-02249-0 40383803 PMC12086256

[64] Yang M, Cheng S, Gu H, et al. Rpl6 interacts with HMGCS1 to stabilize HIF-1α by promoting cholesterol production in hepatocellular carcinoma. Adv Sci. 2025;12(38):e01373. doi: 10.1002/advs.202501373 PMC12520468 40650669

[65] Fu R, Du W, Ding Z, et al. Hif-1α promoted vasculogenic mimicry formation in lung adenocarcinoma through NRP1 upregulation in the hypoxic tumor microenvironment. Cell Death Dis. 2021;12(4):394. doi: 10.1038/s41419-021-03682-z 33850110 PMC8044151

